# Association between care coordination tasks with non-VA community care and VA PCP burnout: an analysis of a national, cross-sectional survey

**DOI:** 10.1186/s12913-021-06769-7

**Published:** 2021-08-13

**Authors:** Eric A. Apaydin, Danielle E. Rose, Michael R. McClean, Elizabeth M. Yano, Paul G. Shekelle, Karin M. Nelson, Susan E. Stockdale

**Affiliations:** 1 Center for the Study of Healthcare Innovation, Implementation and Policy, VA Greater Los Angeles Healthcare System, 11301 Wilshire Blvd., MC 152, Bldg. 206 Rm. 252, Los Angeles, CA 90073 USA; 2 RAND Corporation, Santa Monica, CA USA; 3 Department of Health Policy & Management, Fielding School of Public Health, University of California, Los Angeles, Los Angeles, CA USA; 4 Department of Medicine, David Geffen School of Medicine, University of California, Los Angeles, Los Angeles, CA USA; 5 Seattle-Denver Center of Innovation, VA Puget Sound Health Care System, Seattle, WA USA; 6 Department of Psychiatry and Biobehavioral Sciences, David Geffen School of Medicine, University of California, Los Angeles, Los Angeles, CA USA

**Keywords:** Burnout, Primary care, Care coordination, Healthcare workforce

## Abstract

**Background:**

The scope of care coordination in VA primary care increased with the launch of the Veterans Choice Act, which aimed to increase access through greater use of non-VA Community Care. These changes may have overburdened already busy providers with additional administrative tasks, contributing to provider burnout. Our objective was to understand the role of challenges with care coordination in burnout. We analyzed relationships between care coordination challenges with Community Care reported by VA primary care providers (PCPs) and VA PCP burnout.

**Methods:**

Our cross-sectional survey contained five questions about challenges with care coordination. We assessed whether care coordination challenges were associated with two measures of provider burnout, adjusted for provider and facility characteristics. Models were also adjusted for survey nonresponse and clustered by facility. Trainee and executive respondents were excluded. 1,543 PCPs in 129 VA facilities nationwide responded to our survey (13 % response rate).

**Results:**

51 % of our sample reported some level of burnout overall, and 46 % reported feeling burned out at least once a week. PCPs were more likely to be burned out overall if they reported more than average challenges with care coordination (odds ratio [OR] 2.04, 95 % confidence interval [CI] 1.58 to 2.63). These challenges include managing patients with outside prescriptions or obtaining outside tests or records.

**Conclusions:**

VA primary care providers who reported greater than average care coordination challenges were more likely to be burned out. Interventions to improve care coordination could help improve VA provider experience.

## Background

The Veterans Health Administration (VA) is the nation’s largest integrated healthcare organization, traditionally financing and delivering care within a single system [1]. However, in 2014, the VA Choice Act relaxed certain requirements (among them wait times and distance from the nearest clinic) for Veterans to receive care outside of the VHA [2, 3]. Problems with care coordination between VA and non-VA Community Care under the Choice Act are well-documented [4–6], but it is unclear how these problems affected provider burnout. This increase in non-VA care may have posed unique challenges for VA primary care providers (PCPs; physicians, nurse practitioners, and physician assistants), as they increasingly coordinated care outside of the VA system.

Care coordination, as defined by an Agency for Healthcare Research and Quality review of systematic reviews, is “the deliberate organization of patient care activities between two or more participants (including the patient) involved in a patient’s care to facilitate the appropriate.

delivery of health care services” [7]. The practice has been consistently associated with the delivery of higher quality care. Care coordination increases continuity of care, decreases mortality and hospital admissions, and improves symptoms across a wide range of indications[7]. However, the costs of care coordination are unclear – evidence is mixed on monetary savings of coordination efforts, and their effects on provider and staff time and well-being are largely unknown. One multi-site evaluation of non-provider care coordinators in behavioral health clinics demonstrated high job satisfaction and only moderate emotional exhaustion burnout among these staff [8]. It is unclear if these attitudes extend to providers who conduct care coordination as a portion of their job duties. Care coordination, if well-executed, could be associated with less burnout among providers by increasing efficiency and quality in care delivery. Or, the process could overburden already busy providers, and increase their burnout.

Increased coordination efforts with non-VA care may also disrupt the patient-centered medical home (PCMH; or Patient Aligned Care Team [PACT] in VA) model that all VA PCPs work under. The effects of PCMH on provider experience are mixed. Early work suggested that the PCMH model may improve provider well-being [9], while later work confirmed that the model can increase provider burnout after initial implementation [10–12]. In the VA, challenges with PCMH model fidelity, task sharing [13, 14], and staffing [13, 15] have also been linked to increased provider burnout. However, the VA PCMH model was associated with lower provider burnout when the model was well-implemented [16] and participating providers were engaged in quality improvement efforts [17]. Increasing patient access to non-VA Community Care may unintentionally strain the VA PCMH model by increasing provider workload and by partially negating the efficiency of the integrated care model. Increased staffing and resources may be necessary to support these care coordination demands.

Burnout is a chronic, widespread occupational phenomenon that affects millions of healthcare workers in the United States (US), including 23–60 % of physicians [18]. Burnout is not only damaging to individual workers, but healthcare systems as well. Organizations with burned out workers can experience higher turnover, lower productivity, lower perceived quality of care, and worse patient satisfaction [19–21]. In addition, increased burnout in primary care may aggravate existing shortages in the PCP workforce [22, 23].

Our study is an analysis of the relationship between care coordination demands among VA PCPs with non-VA VA Community Care providers and VA PCP burnout. We seek to understand how challenges with care coordination are related to provider burnout, and whether specific care coordination tasks are larger contributors to decreased well-being. Understanding these relationships will aid in the future development of strategies to deploy care coordination practices that increase provider well-being and the delivery of high-quality care.

## Methods

### Design and Participants

The annual Patient Aligned Care Team (PACT) Survey was fielded to all VA PCPs (physicians, nurse practitioners, and physician assistants) across the US in 2018. Potential respondents were invited by email to complete the anonymous online survey, and non-responders were sent reminders approximately every two weeks (for up to four total reminders). Specialty care providers and non-providers (e.g., nurses, clerks, etc.) were excluded from the sample. Provider trainees and executives or senior executives were also excluded to focus on trained frontline staff without extra administrative control over their practice. This analysis, conducted as a non-research evaluation, and publication were approved by the VA Office of Primary Care.

### Measures

We assessed burnout overall and by frequency of emotional exhaustion burnout symptoms using previously validated single item measures [24, 25]. Respondents rated their overall burnout level by choosing one of the following responses: “I enjoy my work. I have no symptoms of burnout;” “Occasionally I am under stress, and I don’t always have as much energy as I once did, but I don’t feel burned out;” “I am definitely burning out and have one or more symptoms of burnout, such as physical and emotional exhaustion;” “The symptoms of burnout that I’m experiencing won’t go away. I think about frustration at work a lot;” or “I feel completely burned out and wonder if I can go on. I am at the point where I may need some changes or may need to seek help of some sort.” Frequency of burnout symptoms were rated as: never; a few times a year or less; once a month or less; a few times a month; once a week; a few times a week; or every day. In multivariable analyses, binary outcome variables indicating burnout were created for both overall burnout (completely burned out/one or more symptoms of burnout/occasional symptoms of burnout vs. occasional stress/enjoy work) and frequency of burnout (every day/a few times a week/once a week vs. a few times a month/once a month or less/a few times a month/a few times a year or less/never). The binary cut points for overall burnout and burnout frequency were previously validated between the two variables in a study of VA primary care healthcare workers [24]. For burnout frequency, the cut points were validated in studies of surgeons, medical residents, and medical students, in their association with suicidality, perceiving a major medical error, thoughts of dropping out of medical school, endorsing dishonest behavior, and disagreeing with altruistic attitudes [25].

Challenges with care coordination were assessed with five questions on different tasks. Respondents rated their challenge level (I don’t know; not at all challenging; slightly challenging; somewhat challenging; moderately challenging; extremely challenging) with: fielding calls or requests from patients who can’t reach their Community Care providers; fielding calls or requests from patients attempting to schedule routine tests (e.g., imaging) with Community Care providers in a clinically appropriate time frame; managing care of patients who obtained new prescriptions from Community Care providers to be filled by VA pharmacy; managing care of patients who had reduced or eliminated opioid use but obtained new prescriptions from Community Care providers; obtaining outside tests and medical records performed by Community Care providers. The measures were developed in response to an expert panel, composed of healthcare stakeholders (including patients, providers, purchasers,

payers, policy makers, product makers, and principal investigators) [26], run by the RAND Corporation to study access management in VA primary care. Among their recommendations, panel members endorsed the routine evaluation of care provided outside of the VA as an urgent or important goal [27]. These care coordination measures were developed for in response to this recommendation.

Responses to measures were dichotomized into extremely/moderately challenging vs. not at all/slightly/somewhat challenging to aid in analysis and interpretation of results. We deleted cases with missing or “don’t know” responses. Control variables included provider gender, age, race, ethnicity, supervisor status, PACT team tenure, VA tenure, PACT team type (e.g., general, women’s health, etc.), time spent in daily huddles, agreement on receipt of adequate education and training for scope of practice, PACT team staffing level (full staffing vs. less than full staffing), loss of PACT team staff (any in the past 12 months), facility type (VA Medical Center vs. Community-Based Outpatient Clinic), primary care provider-to-staff ratio, and observed-to-expected panel size ratio (i.e., actual panel size divided by VA-recommended panel size). Many of these control variables were used in previous analyses of burnout in VA primary care using the 2014 wave of this survey [15, 28].

### Analyses

Distributions of each variable were examined before analysis. We grouped challenges with care coordination tasks through factor analysis and varimax rotation. All five challenge variables loaded onto one factor (Cronbach’s alpha: 0.84). Multivariable, multilevel logistic regression models estimated relationships between each binary measure of burnout and the factor challenge variable or all challenge variables. Models also included control variables, non-response weights (adjusted for response by facility), and facility-level clustering.

## Results

Overall, 1,543 healthcare providers in 129 facilities responded to the survey, including 1,056 physicians (MD or DO), 397 nurse practitioners, and 89 physician assistants (one respondent declined to state their profession; Tables 1 and 13 % response rate). Nearly 51 % of respondents stated that they experienced burnout, and 46 % reported feeling burned out at least once a week. Other sample characteristics are listed in Table 1. Over half of respondents (Table 2) found two care coordination tasks to be moderately or extremely challenging: obtaining outside tests and medical records from Community Care providers (66.9 %); and managing patients who have received new prescriptions from Community Care providers (55.0 %). On average, participants found care coordination tasks to be somewhat-to-moderately challenging (mean 3.40, standard deviation 1.02, range 1 to 5).

**Table 1:** Characteristics of National Sample of VA Primary Care Providers (n = 1543)

Characteristic	n (%)
*Provider type*	
Physician (MD or DO)	1056 (68.5)
Nurse practitioner	397 (25.8)
Physician assistant	89 (5.8)
*Gender*	
Male	541 (38.2)
Female	876 (61.8)
*Age*	
< 25 years old	1 (0.1)
25–29 years old	9 (0.6)
30–39 years old	176 (12.5)
40–49 years old	355 (25.1)
50–59 years old	485 (34.3)
60 + years old	388 (27.4)
*Race*	
White	1001 (71.5)
Black or African American	55 (3.9)
American Indian or Alaskan Native	9 (0.6)
Asian	240 (17.1)
Native Hawaiian or other Pacific Islander	6 (0.4)
Two or more races	89 (6.4)
*Ethnicity*	
Hispanic or Latino	106 (7.4)
Not Hispanic or Latino	1335 (92.6)
*Years in VA practice*	
< 5 years	619 (42.0)
5 + years	855 (58.0)
*Years in current PACT team*	
<=2 years	100 (6.8)
> 2 years	1369 (93.2)
*Supervisory level*	
None	604 (40.9)
Team Lead (informal)	667 (41.2)
First Line Supervisor	130 (8.8)
Manager	76 (5.2)
*Time spent in daily PACT huddles*	
Do not huddle	219 (14.8)
<=15 min	769 (52.1)
16–30 min	353 (23.9)
> 30 min	136 (9.2)
*Received adequate education/training to function at top of scope of practice*	
Disagree	121 (8.2)
Somewhat disagree	123 (8.3)
Neither agree nor disagree	184 (12.5)
Somewhat agree	261 (17.7)
Agree	764 (51.7)
Don’t know	24 (1.6)
*PACT team is fully staffed*	
Yes	786 (58.7)
No	554 (41.3)
*PACT team had changes or loss in staff over the past 12 months*	
Yes	986 (68.1)
No	462 (31.9)
*PACT team type*	
Primary care	1207 (81.8)
Women’s health	116 (7.9)
Home-based primary care	78 (5.3)
Other	74 (5.0)
*Work at a VA Medical Center*	
Yes	758 (53.1)
No	669 (46.9)
	**M (SD)**
*Primary care provider-to-staff ratio*	2.46 (0.62)
*Observed-to-expected panel size ratio*	0.77 (0.16)
	**n (%)**
*Overall burnout*	
No burnout; enjoy work	197 (13.1)
No burnout; occasional symptoms of stress	529 (35.9)
One or more symptoms of burnout	423 (28.6)
Constant symptoms of burnout	183 (12.4)
Completely burned out	145 (9.8)
*Frequency of burnout*	
Never	101 (6.8)
A few times a year or less	262 (17.7)
Once a month or less	183 (12.4)
A few times a month	246 (16.7)
Once a week	116 (7.9)
A few times a week	310 (21.0)
Every day	259 (17.5)

Note: Responses from providers, excluding trainees, executives, and senior executives; values are missing for some characteristics; column percentages were calculated with denominators of only non-missing values for each characteristic. Abbreviations: *M* mean; *SD* standard deviation; *PACT* patient-aligned care team; *VA* Veterans Health Administration

**Table 2:** National VA Primary Care Provider-Reported Challenges with Elements of Care Coordination

Care coordination element	Provider-reported challenges (n [row % of complete responses]) (n = 1477)					
	**Missing**	**Not challenging**	**Slightly challenging**	**Somewhat challenging**	**Moderately challenging**	**Extremely challenging**
Managing patients who have received new prescriptions from Community Care providers	44	108 (7.54)	250 (17.45)	286 (19.96)	354 (24.70)	435 (30.36)
Managing patients who had reduced/eliminated opioid use but received new opioid prescriptions from Community Care providers	116	253 (18.59)	289 (21.23)	216 (15.87)	229 (16.83)	374 (27.48)
Fielding requests from patients attempting to obtain routine tests from Community Care providers	171	214 (16.39)	244 (18.68)	265 (20.29)	246 (18.84)	337 (25.80)
Obtaining outside tests and medical records from Community Care providers	17	40 (2.74)	153 (10.48)	291 (19.93)	383 (26.23)	593 (40.62)
Fielding requests from patients who can’t reach Community Care providers	174	253 (19.42)	219 (16.81)	247 (18.96)	255 (19.57)	329 (25.25)

Note: Responses from providers, excluding trainees, executives, and senior executives

In multivariable analyses, providers reporting moderate-to-extreme challenges with obtaining outside tests and medical records from Community Care providers (odds ratio [OR] 1.40, 95 % confidence interval [CI] 1.03 to 1.92; Table 3) or managing patients with new prescriptions from Community Care providers (OR 1.83, 95 % CI 1.34 to 2.51) were significantly more likely to report being burned out overall. Providers reporting moderate-to-extreme challenges managing patients with new prescriptions from Community Care providers (OR 1.57, 95 % CI 1.14 to 2.15) were also significantly more likely to be burned out at least once a week. More than average challenges with care coordination tasks overall (as measured by the factor variable) were also related to overall burnout (OR 2.04, 95 % CI 1.58 to 2.63) and experiencing symptoms of burnout at least once a week (OR 1.80, 95 % CI 1.38 to 2.35).

**Table 3:** Odds of Burnout by Challenges with Care Coordination Tasks

Care Coordination Element	Burnout, overall(*n* = 1223)	Burnout, once a week+(*n* = 1227)
	**OR (95 % CI)**	**OR (95 % CI)**
*Managing patients who have received new prescriptions from Community Care providers*		
Not/somewhat/slightly challenging	Ref	Ref
Moderately/extremely challenging	1.83 (1.34–2.51)*	1.57 (1.14–2.15)*
*Managing patients who had reduced/eliminated opiod use but received new opiod prescriptions from Community Care providers*		
Not/somewhat/slightly challenging	Ref	Ref
Moderately/extremely challenging	0.85 (0.62–1.15)	0.86 (0.63–1.17)
*Fielding requests from patients attempting to obtain routine tests from Community Care providers*		
Not/somewhat/slightly challenging	Ref	Ref
Moderately/extremely challenging	0.98 (0.71–1.38)	1.15 (0.81–1.63)
*Obtaining outside tests and medical records from Community Care providers*		
Not/somewhat/slightly challenging	Ref	Ref
Moderately/extremely challenging	1.40 (1.03–1.92)*	1.14 (0.83–1.56)
*Fielding requests from patients who can’t reach Community Care providers*		
Not/somewhat/slightly challenging	Ref	Ref
Moderately/extremely challenging	1.30 (0.92–1.84)	1.37 (0.96–1.94)

Note: Analytic sample was restricted to non-trainee providers who are not executives or senior executives. Models controlled for provider gender, age, race and ethnicity, supervisor status, PACT team tenure, VA tenure, PACT team type, time spent in daily huddles, agreement on receipt of adequate education and training for scope of practice, an of indicator of full PACT team staffing, an indicator for any loss of PACT team staff in the past 12 months, facility type (VA Medical Center vs. Community-Based Outpatient Clinic), provider-to-staff ratio, and observed-to-expected panel size ratio. Models also included sampling (non-response) weights and were clustered by facility. Abbreviations: *CI* confidence interval; *OR* odds ratio; *PACT* patient-aligned care team; *VA* Veterans Health Administration; *: *p* < 0.05 for moderately/extremely challenging vs. not/somewhat/slightly challenging

## Discussion

Challenges in coordinating care with non-VA providers may increase burnout among VA PCPs. Over half of VA PCPs reported overall burnout, and over 40 % reported experiencing symptoms of burnout at least once a week. PCPs that found care coordination tasks with non-VA Community Care providers challenging were more likely to be burned out. Challenges with managing patients with outside prescriptions and obtaining records or tests from outside providers were also specifically linked to PCP burnout.

Physicians in non-VA settings spend 20 % to over 50 % of their day on administrative and nonclinical tasks, like care coordination [29–31]. Time spent coordinating care is not as well studied, but is estimated to make up over a third of a physicians’ nonclinical time [29]. Care coordination or administrative work have not previously been linked to burnout.

VA PCPs may have experienced burnout and increased challenges with care coordination, because of use of third-party administrators under the Choice Act, as has been previously described by Sayre et al. and Tsai et al. [5, 6] The recent passage of the MISSION Act (which reforms the Choice Act) may reduce some of these challenges by returning more control of care coordination back to the VA.

While the VA functions as a single-payer, integrated healthcare delivery system, most healthcare entities in the US operate in multi-payer and non-integrated environment. The multi-payer/non-integrated delivery healthcare system in the US, which VA PCPs must interact with through Community Care, involves a much greater expenditure on administrative costs like care coordination than countries with a single- or more tightly regulated multi-payer systems [32–34]. Pozen and Cutler estimated that the US spends 39 % more than Canada on healthcare administration [32], while Himmelstein and colleagues estimated that the US spends 2–4 times as much as Canada, France, Germany or the Netherlands on administrative costs [33]. Coordinating care within a multi-payer/non-integrated delivery system environment may be inherently complex and likely to lead to burnout.

Our findings suggest that the VA would benefit from avoiding or reducing the administrative burden of care coordination between VA PCPs and non-VA Community Care providers. One solution is to encourage the use of virtual care by veterans previously limited by time or distance from using in-person VA care. Virtual care has shown promise in the VA. A VA video-enabled tablet program elicited high patient satisfaction [35], saved patient time and money [36], and increased access to care [37]. Virtual care was also greatly ramped up in the Houston VA Medical Center after Hurricane Harvey [38], and in private healthcare systems during the COVID-19 pandemic [39]. After the current crisis, it is possible that some veterans will permanently adopt the use of virtual care. Another solution is to hire more dedicated care coordination staff. The MISSION Act has consolidated community care coordination under the VA Office of Community Care and every VA Medical Center now has dedicated staff to coordinate care [40]. Previous research suggests that dedicated administrative staff is preferred by providers [41] and that these staff have high job satisfaction and only moderate burnout [8], implying that care coordination work may be more manageable as a sole rather than added job responsibility.

In non-VA settings, increasing integrated care may decrease challenges with care coordination, and therefore burnout. PCPs in countries that employ the “Beveridge model” (named after William Beveridge, the creator of the UK’s National Health Service) [42] with an integrated national payer and delivery system, like the UK, New Zealand, and Norway, report better care coordination with specialists over medications or care plans compared to the US [43]. Norway employs an integrated national electronic healthcare record (EHR) system, that all relevant stakeholders, including PCPs, hospitals, and others, can access [44]. The UK and New Zealand have less multi-stakeholder integration with their national EHR systems, but countries both explicitly target care coordination in their regulation of PCPs, specialists, and other healthcare professionals [45, 46]. In the US, there is some evidence that more integrated care between primary care physicians and one type of specialist (behavioral health providers) is associated with lower physician burnout [47]. More integrated payment and delivery systems, with better care coordination, may be key to reducing PCP burnout.

This study has several limitations: (1) the response rate was low; (2) the analysis was cross-sectional, so the causal relationship between care coordination (or some mediating factor) and burnout cannot be determined; (3) frequencies of care coordination tasks were not measured, so it is unclear if some tasks were more challenging or simply more frequent; and (4) both measures of burnout were only single items rather than multi-item scales. However, these limitations are offset by several strengths, including use of data from a national survey and analysis of multiple measures of burnout from the same survey.

## Conclusions

In summary, we found that challenges with care coordination with non-VA Community Care providers were linked to burnout among VA PCPs. Encouraging veterans to seek virtual care within the VA or hiring dedicated care coordinators may reduce these challenges, and, as a result, provider burnout as well. In non-VA healthcare settings, a shift towards more integrated care could also reduce burnout and challenges with care coordination. As the VA continues with implementation of the MISSION Act and its continued shift towards virtual care during the COVID-19 pandemic, future research should evaluate implications of these changes over time as well as local innovations in care coordination staffing on burnout.

## Data Availability

The data that support the findings of this study are available from the VA Office of Primary Care, but this data is not publicly available outside of the VA. Data are however available for VA employees from the authors upon reasonable request and with permission of the VA Office of Primary Care.
